# Trends and risk factors of multidrug-resistant hospital-acquired infections in a Tunisian university hospital: repeated point prevalence surveys, 2023–2025

**DOI:** 10.1017/ash.2026.10424

**Published:** 2026-06-04

**Authors:** Salma Balhi, Sameh Boughattas, Mohamed Ben Rejeb, Asma Ben Cheikh, Nihel Haddad, Abdelhalim Trabelsi, Hela Ghali, Houyem Said Laatiri

**Affiliations:** 1 Department of Preventive and Community Medicine, Sahloul University Hospital, University of Sousse, Faculty of Medicine of Sousse, Sousse, Tunisia; 2 Laboratory of Microbiology, Sahloul University Hospital, Sousse, Tunisia, University of Monastir, Faculty of Pharmacy of Monastir, Tunisia

## Abstract

**Objective:**

To assess temporal trends in hospital-acquired infections (HAIs) prevalence, antimicrobial resistance (AMR) patterns, and associated risk factors of multidrug resistance (MDR)-related HAI in a Tunisian hospital between 2023 and 2025.

**Design:**

Repeated point-prevalence surveys (PPSs).

**Setting:**

Sahloul University Hospital, Sousse, Tunisia.

**Patients:**

All patients hospitalized for more than 48 hours during each surveys.

**Methods:**

Data on HAI prevalence, isolated pathogens, and AMR profiles were collected through three PPSs conducted annually from 2023 to 2025. HAIs were defined according to the criteria of the Centers for Disease Control and Prevention. MDR was defined as nonsusceptibility to at least one agent in three or more antimicrobial classes.

**Results:**

From 2023 to 2025, 126 pathogens were responsible for 101 HAIs among 752 surveyed patients. The overall HAI prevalence was 10.9%, with no significant difference between the survey years (trend *P* = .430). Bloodstream infections were the most common type of HAI. Microbiological documentation was available for 94% of cases. Gram-negative bacteria predominated (72.2%) with Klebsiella pneumoniae (15%) as the leading pathogen. Overall, 41.2% of isolates were classified as MDR, with no significant trend over time (trend *P* = .068). Enterobacterales resistant to third-generation cephalosporins accounted for 14.3% (18/126) and carbapenem-resistant Enterobacterales for 7.9% (10/126).

Prolonged hospital stay (>8 d) and ICU admission were independently associated with MDR-related HAIs (adjusted OR = 15.759, 95% CI: 3.691–67.285, *P* < .001 and OR = 3.612, 95% CI: 1.584–8.237, *P* = .002).

**Conclusions:**

Repeated PPSs are an effective surveillance strategy for monitoring HAIs and AMR. These findings support the implementation of targeted antimicrobial stewardship and enhanced infection control interventions in Tunisian hospitals.

## Introduction

Hospital-acquired infections (HAIs) represent a major global health issue, leading to significant morbidity and increased antimicrobial resistance (AMR).^1^ HAIs are defined as infections that occur 48 hours or more after hospital admission, excluding those present or incubating at the time of admission.^2^ They are considered important indicators of patient safety in healthcare settings.^3^ The World Health Organization (WHO) estimates that 7% of hospitalized patients in high-income countries and up to 10% in low-and middle-income countries will acquire at least one HAI during their hospital stay.^4^ In Tunisia, the 2012 national point prevalence survey (PPS) reported an overall HAI prevalence of 6.7%.^5^

At the same time, AMR represents a major public health challenge, largely driven by the selective pressure resulting from the inappropriate use of antibiotics in humans and animals.^6^ Consequently, many HAIs caused by common bacterial pathogens can no longer be treated with first-line antibiotics.^7^ In Tunisia, the extensive use of antibiotics has contributed to the increased incidence rates of AMR bacteria in both hospital and community settings.^8^

Between 2010 and 2020, the global annual incidence of hospital-associated infections resistant to antibiotics was estimated at 136 million cases.^9^ In response to this threat, the WHO published a list of priority pathogens, including *Enterococcus faecium*, *Staphylococcus aureus, Klebsiella pneumoniae, Acinetobacter baumannii*, *Pseudomonas aeruginosa, Enterobacter* spp., categorizing them according to the urgency of the need for new antimicrobial treatments.^6^

In Tunisia, although the growing number of studies addressing HAIs,^10–12^ data on microbiological profiles and AMR patterns among patients with HAIs remain limited. Most existing studies either report limited microbiological documentation of HAI (34.6%)^5^ or do not analyze AMR patterns among these patients.^10–12^

Studies conducted in developed countries have identified several factors associated with an increased risk of MDR bacterial infections. These factors include the intrinsic virulence of the microorganism, inappropriate or excessive use of antibiotics, environmental conditions, and patient-related characteristics.^13,14^ However, because patient profiles and hospital infrastructures vary between countries, these findings may not be directly applicable to the Tunisian context. Therefore, the availability of regional and local data is essential for accurately identifying and understanding the risk factors associated with MDR infections.

This study aimed to assess temporal trends in HAI prevalence, AMR patterns, and associated risk factors of MDR-related HAI at a Tunisian university hospital between 2023 and 2025.

## Methods

### Study design and study population

Three annual PPSs were conducted at Sahloul university hospital in April 2023, April 2024, and April 2025. All surveys followed the same methodology, using a standardized data collection form.

Sahloul University Hospital was founded in 1992 in Sousse, central Tunisia, with a surgical focus. In 2025, it recorded about 330,000 outpatient consultations and 26,000 admitted patients. HAI surveillance using PPS methodology has been conducted since 1991. Since 2012, data have been managed according to a local surveillance protocol using a standardized data entry form.

This study included all patients who developed HAIs 48 hours or more after hospital admission during the prevalence surveys. Patients admitted to emergency and haemodialysis units were excluded due to their short length of stay.

Ethical approval was obtained from the Ethical Review Committee of Sahloul Hospital, and all procedures were conducted in accordance with the principles of the Declaration of Helsinki. All patient information was treated confidentially, and data were analyzed anonymously to ensure privacy protection.

## Case definition

### Hospital-acquired infections

HAIs were defined according to the Centers for Disease Control and Prevention/National Healthcare Safety Network (CDC/NHSN) criteria as infections occurring more than 48 hours after hospital admission, excluding those present or incubating at the time of admission.^2^ The main infection types included common types of HAIs: urinary tract infection (UTI), respiratory tract infection (RTI), bloodstream infection (BSI), surgical site infection (SSI), skin and soft tissue infection.^2^

When a patient presented with multiple infection sites, each infection was recorded separately An HAI. was considered active if the patient showed clinical symptoms of infection or was receiving antimicrobial therapy for an infection on the day of the survey, with symptom onset occurring at least 48 hours after hospital admission.

*Medical device use* was defined as the presence of at least one of the following devices at survey day: central venous catheter, invasive mechanical ventilation, urinary catheter, or peripheral vascular catheter.^2^

*Multidrug-resistant pathogens* were defined as microorganisms exhibiting acquired non-susceptibility to at least one agent in three or more antimicrobial categories.^15^ Surveillance of MDR bacteria at Sahloul hospital followed “WHO Priority Pathogens List” and were categorized into five groups^6^:

- *Acinetobacter baumannii* resistant to imipenem,
- Enterobacterales resistant to third-generation cephalosporins and/or carbapenems,
- *Pseudomonas aeruginosa* resistant to carbapenems (imipenem) and/or third-generation cephalosporins (ceftazidime)
- Methicillin-resistant *Staphylococcus aureus* (MRSA),
- E*nterococcus faecium resistant to vancomycin* (VRE).

## Microbiological methods and antibiotic susceptibility

Microbiological identification and antimicrobial susceptibility testing were performed in the microbiology laboratory of Sahloul hospital. Clinical specimens included blood, urine, protected respiratory distal specimens, sputum, pus and catheters.

Species identification was performed using conventional methods, colony morphology, Gram staining, appropriate biochemical tests, and the VITEK-2 automated system (VITEK-2 Biomérieux, France).

Antimicrobial susceptibility testing was performed according to the CA-SFM/EUCAST (European Committee on Antimicrobial Susceptibility Testing) recommendations applicable for the respective survey years.^16^

## Data collection

The study was conducted by a multidisciplinary team from the Department of Preventive and Community Medicine, comprising hygienist technicians, nurses, and medical doctors. This team was responsible for data collection and overall study implementation. Due to the limited number of investigators, data collection across all hospital wards was conducted over a two-week period. In each ward, data were collected on a single designated day, and each bed was surveyed only once.

Data were collected from multiple sources including medical records, temperature charts, laboratory reports, and radiological findings. Additional information was obtained from attending nurses and doctors as needed.

Data were recorded using the same standardized form across all three surveys. The questionnaire consisted of four structured sections: (1) Demographic data, (2) Clinical data, (3) Extrinsic risk factors, including exposure to invasive devices (central or peripheral intravascular catheter), indwelling urinary catheter, and mechanical ventilation, (4) HAI-related data, including infection site (if documented), responsible microorganism and AMR profile. Only active HAIs present on the survey date were recorded.

## Data analysis

Data were analyzed using IBM Statistical Package for the Social Sciences (SPSS), version 27.0. Descriptive statistics were used to summarize patient demographics, clinical characteristics, and microbiological patterns. Categorical variables were presented as frequencies and percentages, while continuous variables were reported as medians and interquartile ranges (IQRs). The prevalence of HAI was calculated as the number of patients with at least one infection divided by the total number of patients surveyed during each PPS. Comparisons between categorical variables were performed using the *χ* ^2^ test, while continuous variables were compared using the Mann-Whitney *U* test.

The Cochran-Armitage trend test was performed to evaluate changes in the prevalence of HAIs, infection sites, pathogen distribution, and AMR over the study period.

Binary logistic regression analysis was performed to identify independent predictors associated with MDR-related HAIs. Patients with at least one HAI caused by MDR bacteria were coded as 1, whereas those without MDR-related HAIs were coded as 0. Variables with a *P*-value ≤.20 in the univariate analysis were included in the multivariable regression model. Results were reported as odds ratio (OR) with 95% confidence intervals (95% CIs). A two-sided *P*-value <.05 was considered statistically significant for all analyses.

## Flow chart

**Figure 1.**
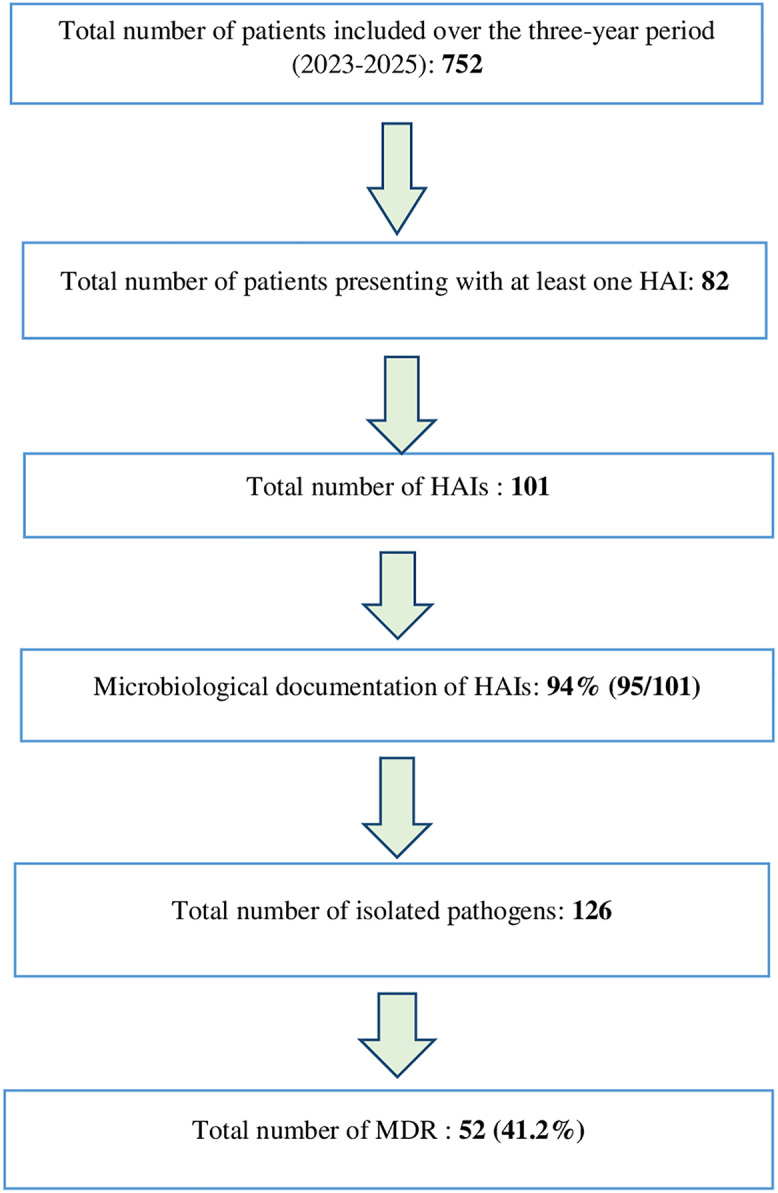
Figure 1 long description. Flow chart of the study.

The flow chart Figure 1 illustrates the key steps involved in our methodological approach.

## Results

### Baseline characteristics of the patients

Overall, 752 patients were included in the three prevalence surveys performed between 2023 and 2025. The median age of all patients was 49 years [IQR = 22–64], with a higher proportion of males (60%). On the survey day, the majority of patients (44.8%) were admitted to medical wards, followed by surgical wards (42.4%), and the intensive care units (12.8%). The median length of stay was 8 days (IQR: 4–15).

Regarding medical device use, a peripheral vascular catheter was present in 61.2% of patients, a urinary catheter in 25.3%, and a central vascular catheter in 10.9% of cases. The proportion of mechanically ventilated patients was 6.0%. Additionally, 31.8% of patients had undergone surgery within the previous 30 days, and 32.0% had received antimicrobial treatment during the past six months Table 1.

**Table 1.** Demographic and clinical characteristics of surveyed patients Table 1 long description.

Characteristics	All patients (N = 752)	Patients without HAI (N = 670)	Patients with HAI (N = 82)	*P*-value
**Sex**				**.844**
*Male*	451 (60.0)	401 (59.9)	50 (61)	
*Female*	301 (40.0)	269 (40.1)	32 (39)	
**Age (median IQR, years)**	49 [22–64]	49 [21–64]	49 [29–64.25]	**.795**
**Ward of admission, n (%)**				**<.001**
*Medical*	337 (44.8)	319 (47.6)	18 (22)	
*Surgical*	319 (42.4)	292 (43.6)	27 (32.9)	
*ICU*	96 (12.8)	59 (8.8)	37 (45.1)	
**Length of stay, median, (interquartile range), days**	8 (4–15)	7 (4–14)	18.5 (12–30)	**<.001**
**Central catheter in place on survey day**				**<.001**
*Yes*, n (%)	82 (10.9)	48 (7.2)	34 (41.5)	
**Peripheral vascular catheter in place on survey day**				**.060**
*Yes*, n (%)	460 (61.2)	402 (60)	58 (70.7)	
**Urinary catheter in place on survey day**				**<.001**
*Yes*, n (%)	190 (25.3)	145 (21.6)	45 (54.9)	
**Ventilator in place on survey day**				**<.001**
*Yes*, n (%)	45 (6.0)	26 (3.9)	19 (23.2)	
**Patient with at least one medical devices at the survey day**				**<.001**
n (%)	518 (68.9)	442 (66.0)	76 (92.7)	
**Patient underwent surgery in the previous 30 days**				**<.001**
*Yes*, n (%)	239 (31.8)	188 (28.1)	51 (62.2)	
**Patient received antimicrobial treatment in the past six months**				**.007**
*Yes*, n (%)	241 (32.0)	204 (30.4)	37 (46.1)	

HAI, hospital-acquired infection, IQR, interquartile range, ICU, intensive care unit, *P*-value: *χ* ^2^ test for categorical variables and Mann Whitney test for continuous variables.

### Prevalence of hospital-acquired infections

Between 2023 and 2025, 82 of 752 patients (10.9%) had at least one HAI, accounting for a total of 101 HAIs. Of these, 32 HAIs were identified in 2023, 31 in 2024, and 38 in 2025. The prevalence of patients with at least one HAI showed a slight but non-significant increase from 10.2% in 2023 to 11.4% in 2025 (*P* for trend = .43). By department, the prevalence of patients with at least one HAI was highest in the ICU (38.5%, 37/96), compared to 6.8% (45/656) in non-ICU wards (combined surgical and medical wards) (*P* < .001).

During the three-year study period, the most frequently occurring types of HAI were BSI (29.7%), followed by RTI (27.7%), UTI (20.8%) and SSI (19.8%) Table 2. The proportion of BSIs showed an increase from 28.1% in 2023 to 34.2% in 2025. However, no significant temporal trend was observed (*P* for trend = .815).

**Table 2.** Prevalence of hospital-acquired infections at Sahloul University Hospital, Tunisia 2023–2025 Table 2 long description.

	2023 (n = 254) n (%)	2024 (n = 243) n (%)	2025 (n = 255) n (%)	Total (n = 752) n (%)	*P* for trend^*^
**Patient with at least one HAI**	26 (10.2)	27 (11.1)	29 (11.4)	82 (10.9)	.430
**All HAIs**	32 (12.6)	31 (12.7)	38 (14.9)	101 (13.4)	.777
**Distribution of infection sites**					
*BSI*	9 (28.1)	8 (25.8)	13 (34.2)	30 (29.7)	.815
*RTI*	8 (25)	8 (25.8)	12 (31.6)	28 (27.7)	.823
*UTI*	6 (18.7)	9 (29)	6 (15.8)	21 (20.8)	.497
*SSI*	8 (25)	6 (19.3)	6 (15.8)	20 (19.8)	.288
*Others*	1 (3.1)	0	1 (2.6)	2 (2)	NT

HAI, hospital-acquired infection, UTI, urinary tract infection, RTI, respiratory tract infection, SSI, surgical site infection BSI, bloodstream infection, Others: skin and soft tissue infection, cardiovascular infection.

* Cochran-Armitage trend test analysis, NT, Not tested low frequency.

### Microbiology documentation

During the study period, microbiological documentation was available for 94% (95/101) of HAIs. The most frequently collected specimens were blood cultures (31.5%), followed by urine (22.1%), protected distal samples (20%), and pus (17.9%).

Overall, 126 strains belonging to 24 distinct species were obtained from infected patients. Gram-negative bacteria predominated, accounting for 72.2% of all isolates. The most common gram-negative pathogens were *Klebsiella pneumoniae* (15%), *Acinetobacter baumannii* (14.3%), and *Escherichia coli* (12.7%). Gram-positive bacteria accounted for 26.2% of isolates, with *Staphylococcus aureus* (11.6%) being the most common species. Fungi were identified in 1.6% of cases. The proportion of gram-negative bacteria decreased from 79% in 2023 to 68.1% in 2025, while Gram-positive bacteria increased from 21% to 29.8%. However, these changes were not statistically significant (*P* for trend = .137 for gram-negative, and *P* for trend = .816 for Gram-positive) (Table 3).

**Table 3.** Distribution of hospital-acquired infection pathogens, in a tertiary hospital in Tunisia, 2023–2025 Table 3 long description.

Pathogens	2023 N = 38 (%)	2024 N = 41 (%)	2025 N = 47 (%)	Total N = 126 (%)	*P* for trend^*^
**Gram-positive bacteria**	8 (21)	11 (26.8)	14 (29.8)	33 (26.2)	.816
*Staphylococcus aureus*	8 (21)	3 (7.3)	3 (6.3)	14 (11.6)	
*Coagulase-negative staphylococci*	0	3 (7.3)	6 (12.7)	9 (7.1)	
*Enterococcus faecalis*	0	2 (4.8)	2 (2.1)	4 (3.3)	
*Enterococcus faecium*	0	2 (4.8)	1 (4.2)	3 (2.5)	
*Others*	0	1 (2.4)	2 (4.2)	3 (2.5)	
**Gram-negative bacteria**	30 (79)	29 (70.8)	32 (68.1)	91 (72.2)	.137
*Klebsiella pneumoniae*	8 (21)	4 (9.7)	7 (14.9)	19 (15)	
*Acinetobacter b aumannii*	3 (7.9)	8 (19.5)	7 (14.9)	18 (14.3)	
*Escherichia coli*	7 (18.4)	6 (14.6)	3 (2.4)	16 (12.7)	
*Pseudomonas aeruginosa*	5 (13.1)	3 (7.3)	6 (12.7)	14 (11.1)	
*Enterobacter cloacae*	3 (7.9)	1 (2.4)	4 (8.5)	8 (6.3)	
*Stenotrophomonas maltophilia*	0	1 (2.4)	3 (6.4)	4 (3.1)	
*Proteus mirabilis*	1 (2.6)	1 (2.4)	1(2.1)	3 (2.4)	
*Others*	3 (7.9)	5 (12.2)	1(2.1)	9(7.1)	
**Fungi**	0 (0)	1 (2.4)	1 (2.1)	2 (1.6)	**NT**
*Candida*	0	1 (2.4)	1 (2.1)	1 (0.8)	

Percentages are calculated based on the total of each column*. Overall percentages are calculated on total isolates (N = 126).

* Cochran-Armitage trend test analysis, NT, not tested (low frequency).

Of all MDR isolates (41.2%, 52/126), 30.7% were obtained from protected distal respiratory specimens, 26.9% from blood cultures, and 25.0% from pus samples. The proportion of HAIs caused by MDR bacteria decreased from 57.9% in 2023 to 40.4% in 2025. Nevertheless, this change was not statistically significant (*P* for trend = .068) Table 4.

**Table 4.** Distribution of multidrug-resistance pathogens isolated from hospital-acquired infections, 2023–2025 Table 4 long description.

	2023 (N = 38) n (%)	2024 (N = 41) n (%)	2025 (N = 47) n (%)	Total 2023–2025 (N = 126) n (%)	*P* for trend^*^
***Total Gram-positive MDR***	2 (5.2)	0	2 (4.2)	4 (3.1)	NT
*Staphylococcus aureus*, MRSA	2 (5.2)	0	1 (2.1)	3 (2.4)	–
*Enterococcus faecium*, VRE	0	0	1 (2.1)	1 (0.8)	–
***Total Gram-negative MDR***	20 (52.6)	11 (26.8)	17 (36.1)	48 (38)	.074
*Acinetobacter baumannii*, MDR	3 (7.9)	6 (14.6)	6 (12.8)	15 (11.9)	–
*Pseudomonas aeruginosa*, MDR	3 (7.9)	0	1 (2.1)	4 (3.1)	–
***Total Enterobacterales resistant to third-generation cephalosporins***	10 (26.3)	2 (4.8)	6 (12.7)	18 (14.3)	.050
***Total Enterobacterales resistant to carbapenems***	4 (10.5)	2 (4.8)	4 (8.5)	10 (7.9)	.386
Enterobacterales, resistant to third-generation cephalosporins and/or carbapenems	–	–	–	–	–
*Klebsiella pneumoniae*	6 (15.8)	3 (7.3)	6 (12.8)	15 (11.9)	
*Escherichia coli*	6 (15.8)	1 (4.8)	1 (2.1)	9 (7.1)	
*Enterobacter cloacae*	1 (2.6)	0	3 (6.4)	4 (3.1)	
*Proteus mirabilis*	1 (2.6)	0	0	1 (0.8)	
**Total MDR isolates**	22 (57.9)	11 (26.8)	19 (40.4)	52 (41.2)	.068

Percentage are calculated of the total isolates recorded in each column, N, Total number of pathogens targeted by microbiological surveillance, MDR, Multidrug-resistant, MRSA: methicillin resistant S.aureus, VRE, Vancomycin-resistant Enterococci, test de Cochran-Armitage.

* Cochran-Armitage trend test analysis, NT low frequency.

By bacterial family, gram-negative MDR bacteria accounted for the largest share, representing 38% (48/126) of all pathogens isolated. The proportion of Enterobacterales resistant to third-generation cephalosporins was 14.3% (18/126). Carbapenem-resistant Enterobacterales accounted for 7.9% of all isolates (10/126).

Among the identified microorganisms, MDR *Acinetobacter baumannii* and MDR *Klebsiella pneumoniae* were the most prevalent, each accounting for 11.9% (15/52) of isolates, followed by MDR *Escherichia coli* at 7.1% (9/52) (Table 4).

## Factors associated with MDR-related HAI

Overall, 37 patients (4.9%) had HAIs caused by MDR bacteria. In univariate analysis, several factors were associated with MDR-related HAIs, including admission to the ICU (OR = 5.505, 95% CI: 2.526–12.001), prolonged hospital stay greater than 8 days (OR = 22.863, 95% CI: 5.457–95.787), medical device use on the survey day (OR = 8.406, 95% CI: 2.005–35.249), surgical intervention within the past 30 days (OR = 3.366, 95% CI: 1.713–6.613), and antibiotic use in the previous six months (OR = 2.630, 95% CI: 1.351–5.117).

In the multivariate logistic regression model, prolonged hospital stay (>8 d) and ICU admission remained independent predictors of MDR-related HAIs, with an AOR of 15.759 (95% CI: 3.691–67.285, *P* <.001) and 3.612 (95% CI: 1.584–8.237, *P* = .002), respectively (Table 5).

**Table 5.** Factors associated with MRD-HAI patients during the three point prevalence survey at Sahloul University Hospital, 2023–2025 Table 5 long description.

Variables (N = 752)	MDR-HAI, n (%)			COR (95% CI)	AOR (95% CI)	*P*-value
	Yes	No	*P*-value			
**Gender**	–	–	.780	–	–	–
*Male*	23 (3.1)	428 (56.9)	–	0.908 (0.459–1.794)	–	
*Female*	14 (1.9)	287 (38.2)	–	Ref	–	
**Age (years)**	–	–	.716	–	–	–
*>50*	19 (2.5)	389 (51.7)	–	0.885 (0.457–1.714)	–	
*≤50*	18 (2.4)	326 (43.4)	–	Ref		
**Ward of admission**	–	–	<.001	–	–	.646 .002
*Medical*	8 (1.1)	329 (43.8)	–	0.622 (0.251–1.542)	1.280 (0.447–3.666)	
*Intensive care unit*	17 (2.3)	79 (10.5)	–	5.505 (2.526–12.001)	3.612 (1.584–8.237)	
*Surgical*	12 (1.6)	307 (40.8)	–	Ref	Ref	
**Length of stay (days)**	–	–	<.001	–	–	<.001
>8	35 (4.7)	310 (41.2)	–	22.863 (5.457–95.787)	15.759 (3.691–67.285)	
*≤8*	2 (0.3)	405 (53.9)	–	Ref	Ref	
**Diabetes**	–	–	.986	–	–	–
*Yes*	28 (3.7)	173 (23)	–	1.007 (0.466–2.176)	–	
*No*	9 (1.2)	542 (72.1)	–	Ref	–	
**Medical devices use**	–	–	.001	–	–	.067
*Yes*	35 (4.7)	483 (64.2)	–	8.406 (2.005–35.249)	4.117 (0.904–18.740)	
*No*	2 (0.3)	232 (30.9)	–	Ref	Ref	
**Surgical intervention within the past 30 days**	–	–	<.001	–	–	.208
*Yes*	22 (2.9)	217 (28.9)	–	3.366 (1.713–6.613)	1.690 (0.747–3.824)	
*No*	15 (2)	498 (66.2)	–	Ref		
**Antibiotic use in the past 06 months**	–	–	.003	–	–	.189
*Yes*	20 (2.7)	221 (29.4)	–	2.630 (1.351–5.117)	1.621 (0.788–3.334)	
*No*	17 (2.3)	494 (65.7)	–	Ref	–	
**Infection at admission**	–	–	.106	–	–	–
*Yes*	13 (1.7)	168 (22.3)	–	1.764 (0.879–3.540)	–	
*No*	24 (3.2)	547 (72.2)	–	Ref	–	

MDR-HAI, Multidrug-resistant hospital acquired infections, *P* value: significant less than.05, Ref, References classe, Percentages were calculated based on the total effective.

## Discussion

HAIs caused by MDR bacteria represent a worldwide challenge, as they pose serious life-threatening risks.^17^ Preventing these infections is therefore a major priority. Achieving effective prevention requires an understanding of local infection rates, the bacteriological profile and associated AMR patterns.

The overall prevalence of HAIs over the three consecutive years (2023–2025) was 10.9%, which is lower than the rate reported during the COVID-19 pandemic in 2020 (14.9%).^12^ Key strategies include audit and feedback to enhance compliance with best practices, regular staff training, continuous surveillance of HAIs, and Celebration of the World Hand Hygiene Day. Furthermore, established protocols are in place to ensure adherence to standard and contact precautions for patients with MDROs, including the investigation of all reported MDR cases, patient isolation, and reinforcement of hygiene measures. However screening for MDRO in our hospital is not performed systematically due to limited resources. It is mainly carried out when patients are transferred from another healthcare facility to high-risk or critical units such as intensive care, nephrology, and pediatrics.

Globally, HAI prevalence varies considerably between settings. In Africa, a recent meta-analysis including 81,968 patients across 20 countries reported prevalence rates ranging from 1.6% to 90.2%.^18^ In contrast, the European PPS reported a prevalence of 7.1% in 2024.^19^ These disparities may be explained by differences in hospital infrastructure and patient comorbidities.^20^ They may also result from variations in HAI definitions, surveillance methodologies, and data collection procedures.^20^

Overall, the burden of HAI in the ICU remains high, with approximately four in ten ICU patients (38.5%) developing at least one HAI. These findings are consistent with reports from other countries, where the prevalence of HAIs among ICU patients ranges from 15.3% to 24.7%.^10,21,22^ This high prevalence may be explained by patients’ increased vulnerability, the severity of their underlying conditions, and the frequent use of invasive devices.

BSIs represented the most common HAI site across all three surveys, in contrast to other Tunisian studies where RTIs^10,12^ were more prevalent. This observation may be explained by the high device utilization rate (68.9%) observed in our cohort, given the well-established association between vascular devices and catheter-related BSIs. Therefore, strengthening BSI prevention strategies should be prioritized through the implementation of regular audits of practices related to the insertion of central and peripheral vascular catheters in our healthcare settings.

Microbiological documentation was available for 94% of HAI cases, which is higher than proportions reported in previous studies.^5,20^

Over the three-year period, gram-negative bacteria were isolated three times more frequently than Gram-positive bacteria (72.2% vs 26.2%). Similar patterns have been reported in international studies conducted in China,^20^ India,^23^ Canada,^22^ and Saudi Arabia,^21^ where gram-negative bacteria were identified as the leading pathogens responsible for HAIs. Indeed, gram-negative bacteria represent a major therapeutic challenge as they are often associated with severe infections such as pneumonia, BSIs, and UTIs, particularly among critically ill or immunocompromised patients.^24^

*Klebsiella pneumoniae* was the most frequently isolated pathogen across all three surveys, followed by *Acinetobacter baumannii*, *Escherichia coli and Pseudomonas aeruginosa.* These findings are consistent with previous Tunisian studies,^10,12^ in which *Klebsiella pneumoniae* predominated. Nevertheless, they differ from reports where *Escherichia coli* was the leading pathogen.^18,25^ Such discrepancies may reflect variations in patient characteristics, healthcare practices, and local microbial ecology.

Between 2023 and 2025, MDR bacteria accounted for 41.2% of all isolated pathogens, with no statistically significant change over the study period. This stability may reflect limited changes in antimicrobial stewardship policies or infection control measures during this period across the hospital. Preventive management of MDR-related HAIs may involve cohorting of infected patients, strict adherence to standard precautions, and targeted interventions based on transmission routes.^17^

The proportion of carbapenem-resistant Enterobacterales was 7.9% (10/126). Indeed, since 2006, several carbapenemase-producing gram-negative bacteria, along with different carbapenemase variants, have been reported in Tunisia.^26^ Given the limited availability of new antimicrobial agents, continuous monitoring of regional prevalence and resistance profiles is crucial.^26^

At the species level, *Klebsiella pneumoniae* and *Acinetobacter baumannii* were the most frequently identified MDR organisms (11.9% each), followed by *Escherichia coli* (7.1%). These findings are consistent with those of the second national PPS conducted in Tunisia, in which *Acinetobacter baumannii* accounted for 11.7% of all isolates.^5^ Similarly, another Tunisian study investigating long-term trends in MDR gram-negative bacteria between 1999 and 2019 reported a marked increase in carbapenem resistance in *Acinetobacter baumannii*, with imipenem resistance rising from 34.5% in 2008 to 84.2% in 2019.^26^ These findings highlight the growing therapeutic challenges posed by this pathogen in hospital settings.

In multivariate analysis, a prolonged length of hospital stay was identified as an independent predictor of MDR-related HAIs. Prolonged hospitalization is widely recognized as one of the most important risk factors for MDR-related HAIs.^27,28^ This study also revealed that ward type had a statistically significant association with MDR-related HAIs. The ICU showed higher odds for developing MDR infection than other wards. One explanation is that ICUs are generally associated with increased use of broad-spectrum antibiotics, greater exposure to invasive medical devices, and patient vulnerability.^21^ Such data can facilitate the design and implementation of effective antimicrobial stewardship programs. It may also help guide the selection of appropriate empirical antibiotic therapy in patients presenting with risk factors of infections.

## Strengths and limitations

The main strength of this study is that it represents the first Tunisian investigation to assess trends in HAIs and MDR bacteria in a tertiary hospital in Tunisia using three repeated PPS. Moreover, the study achieved a high level of microbiological documentation (94%). Unlike previous Tunisian reports on HAIs, which either did not analyze AMR patterns^10,12^ or reported very low levels of microbiological documentation (36%),^5^ our study offers a more comprehensive picture of the local epidemiology. These findings may also serve as a foundation for future interventional studies aimed at controlling MDRO at the hospital level.

Nevertheless, this study has some limitations. First, repeated prevalence surveys at regular intervals provide valuable comparative data, revealing secular trends of infections and evaluating control programs.^29,30^ However, both underreporting and overreporting of HAI rates may occur in this type of study. To minimize this bias, multiple sources of information were verified during data collection, including medical reports, radiological and laboratory tests, as well as direct interviews with attending doctors. Second, the survey may not be fully representative of all HAI patients in Tunisia due to the single-center design and relatively small sample size. Finally, there is a risk of inconsistent adjudication considering turnover among hospital staff reviewing medical reports. However, standardized training for data investigators was provided within the same department across the three surveys to reduce inconsistencies in data collection.

## Conclusions

Our study revealed a relatively high prevalence of MDR-related HAIs in a tertiary care hospital in Tunisia over the three-year survey period. BSIs were the most frequent site of infection. Admission to the ICU and prolonged length of stay were independently associated with MDR-related HAIs. Promoting the appropriate use of antibiotics, especially in ICU settings, together with strict adherence to standard precautions, may help limit the spread of MDR pathogens in Tunisian hospitals. These findings support the implementation of targeted antimicrobial stewardship and enhanced infection control interventions in Tunisian hospitals.

## Figure 1. Long description

The flow chart outlines the study of hospital-acquired infections (HAIs) over a three-year period from 2023 to 2025. It begins with the total number of patients included, which is 752. Out of these, 82 patients presented with at least one HAI, resulting in a total of 101 HAIs. Microbiological documentation was available for 94% of these HAIs, with 95 out of 101 cases documented. The total number of isolated pathogens identified was 126, with 52 of these being multidrug-resistant (MDR), accounting for 41.2% of the isolated pathogens. The chart visually represents the progression and key statistics of the study on HAIs.

Navigate back to Figure 1..

## Table 1. Long description

The table presents demographic and clinical characteristics of 752 surveyed patients, divided into those without hospital-acquired infections (670 patients) and those with hospital-acquired infections (82 patients). It includes data on sex, age, ward of admission, length of stay, and the presence of various medical devices such as central catheters, peripheral vascular catheters, urinary catheters, and ventilators. The table also notes whether patients underwent surgery in the previous 30 days or received antimicrobial treatment in the past six months. Notable trends include a higher median length of stay for patients with hospital-acquired infections (18.5 days) compared to those without (7 days), and a significantly higher proportion of patients with hospital-acquired infections having central catheters (41.5%) and urinary catheters (54.9%) in place on the survey day.

Navigate back to Table 1..

## Table 2. Long description

The table presents data on the prevalence of hospital-acquired infections (HAI) at Sahloul University Hospital in Tunisia over three years, from 2023 to 2025. It includes the number and percentage of patients with at least one HAI, the total number of HAIs, and the distribution of infection sites. The table has four rows and five columns, with column headers for each year (2023, 2024, 2025) and the total, along with row labels for different infection categories. Notable trends include an increase in bloodstream infections (BSI) from 28.1 percent in 2023 to 34.2 percent in 2025, although no significant temporal trend was observed. The data is presented in percentages and includes categories such as respiratory tract infections (RTI), urinary tract infections (UTI), surgical site infections (SSI), and others.

Navigate back to Table 2..

## Table 3. Long description

The table presents the distribution of hospital-acquired infection pathogens in a tertiary hospital in Tunisia over the years 2023, 2024, and 2025. It includes data on Gram-positive bacteria, Gram-negative bacteria, and fungi, with a total of 126 strains belonging to 24 distinct species. The table has 15 rows and 6 columns, with column headers including the years 2023, 2024, 2025, Total, and P for trend. Row labels include different pathogens such as Staphylococcus aureus, Klebsiella pneumoniae, and Escherichia coli. Notable trends include a decrease in the proportion of Gram-negative bacteria from 79 percent in 2023 to 68.1 percent in 2025, and an increase in Gram-positive bacteria from 21 percent to 29.8 percent. The most common Gram-negative pathogens are Klebsiella pneumoniae, Acinetobacter baumannii, and Escherichia coli, while Staphylococcus aureus is the most common Gram-positive pathogen. Fungi are identified in 1.6 percent of cases. The changes in proportions of Gram-negative and Gram-positive bacteria are not statistically significant.

Navigate back to Table 3..

## Table 4. Long description

The table presents data on the distribution of multidrug-resistance pathogens isolated from hospital-acquired infections over the years 2023, 2024, and 2025. It includes the total number of isolates and their percentages for each year, as well as the overall totals and trends. The table is divided into categories such as Total Gram-positive MDR, Staphylococcus aureus MRSA, Enterococcus faecium VRE, Total Gram-negative MDR, Acinetobacter baumannii MDR, Pseudomonas aeruginosa MDR, Total Enterobacterales resistant to third-generation cephalosporins, Total Enterobacterales resistant to carbapenems, and Total MDR isolates. Notable trends include a decrease in the proportion of hospital-acquired infections caused by MDR bacteria from 57.9% in 2023 to 40.4% in 2025, although this change is not statistically significant.

Navigate back to Table 4..

## Table 5. Long description

The table presents data from a multivariate logistic regression model analyzing factors associated with multi-drug-resistant hospital-acquired infections (MDR-HAI) among patients at Sahloul University Hospital between 2023 and 2025. The table includes variables such as gender, age, ward of admission, length of stay, diabetes, medical devices use, surgical intervention within the past 30 days, antibiotic use in the past six months, and infection at admission. It shows the number and percentage of patients with and without MDR-HAI for each variable, along with crude odds ratios (COR) and adjusted odds ratios (AOR) with 95% confidence intervals (CI) and P-values. Notable findings include prolonged hospital stay greater than eight days and intensive care unit admission as independent predictors of MDR-related HAIs, with AORs of 15.759 and 3.612, respectively. The table highlights significant P-values and odds ratios, indicating strong associations between these factors and MDR-HAI.

Navigate back to Table 5..
